# Dynamic Genetic Changes Reveal: Intra-Lineage Diversity, Not Admixture, Explains *Amaranthus palmeri*’s Success in China

**DOI:** 10.3390/ijms26178128

**Published:** 2025-08-22

**Authors:** Jing-Jing Cao, Hong-Wei Wang, Jian-Guo Fu, Fang-Hao Wan, Jian-Ying Guo, Rui Wang

**Affiliations:** 1 State Key Laboratory for Biology of Plant Diseases and Insect Pests, Institute of Plant Protection, Chinese Academy of Agricultural Sciences, Beijing 100193, China; caojingjing319@163.com (J.-J.C.); wanfanghao@caas.cn (F.-H.W.); guojianying@caas.cn (J.-Y.G.); 2 College of Plant Protection, Henan Agricultural University, Zhengzhou 450002, China; whwcas@163.com; 3 Animal, Plant and Food Inspection Center, Nanjing Customs, Nanjing 210019, China; fujianguo07@163.com

**Keywords:** *Amaranthus palmeri*, biological invasions, multiple introductions, genetic admixture, genetic diversity

## Abstract

Global trade facilitates multiple introductions of alien species, yet the role of genetic admixture between divergent lineages in driving invasion success remains debated. Here, we address this question by analyzing dynamic genetic changes across invasion stages in the dioecious weed *Amaranthus palmeri*, introduced to China from North and South America. Combining chloroplast phylogeography with nuclear genetic analyses, we systematically investigated genetic changes in populations at casual, naturalized, invasive, and dispersal stages. Initial casual populations originated from distinct North and South American lineages, but all established populations (naturalized, invasive, dispersal) retained only North American haplotypes. South American genetic introgression decreased progressively during invasion (from 34% in naturalized to 3% in dispersal populations), accompanied by declining inbreeding coefficients. Established populations exhibited high inter-population crosses within the North American lineage (54–60%), maintaining genetic diversity and overcoming bottlenecks. Our findings demonstrate that invasion success in *A. palmeri* may be driven by gene flow within the North American lineage, rather than admixture between divergent lineages. These findings enhance our understanding of the genetic mechanisms underpinning plant invasions, highlighting lineage-specific management as a critical strategy for controlling invasive populations.

## 1. Introduction

Biological invasions pose a significant and growing threat to global ecosystems and economies, with estimated annual costs exceeding $1.3 trillion [1]. The Anthropocene era, characterized by accelerated international trade, particularly in agricultural commodities, has dramatically increased the rate at which alien plant species are introduced [2]. Crucially, these introductions often involve multiple, genetically distinct source populations [3]. This process leads to genetic admixture within the introduced range, where previously isolated lineages mix [4].

The role of genetic admixture from divergent lineages (hereafter “admixture”) in invasion success is currently debated [5,6]. Most researchers suggest that admixture facilitates invasions [4]. Potential benefits include increased genetic diversity [7], novel genotypes [8], and possibly heterosis (hybrid vigor) [9,10]. However, some studies indicate that admixture can be neutral or even detrimental [11,12]. It might introduce maladaptive gene combinations to reduce fitness [13], thereby hindering initial invasion success. This conflict highlights a critical uncertainty: whether genetic admixture actually facilitates invasion success [14,15].

Resolving this contradiction requires moving beyond static, single-timepoint analyses of established populations. Current studies mainly reconstruct invasion histories in a retrospective manner [16]. Such approaches may fail to capture the dynamic genetic changes occurring across different stages of the invasion process [17]. The invasion continuum involves critical transitions—transportation, introduction, establishment, and spread [18]—leading to the formation of casual (transient), naturalized (self-sustaining), and invasive (expanding and secondly spread) populations [19,20,21,22]. Admixture levels might fluctuate non-linearly during this process due to factors like initial founder effects, subsequent secondary introductions, selection pressures favoring specific hybrid genotypes, or genetic drift during range expansion [4,15]. Therefore, a comprehensive understanding demands comparative analyses of genetic architecture across these distinct invasion stages to distinguish persistent advantages conferred by admixture from transient demographic effects.

Combining analyses of conserved chloroplast DNA (cpDNA) and polymorphic nuclear DNA offers a powerful strategy for examining temporal genetic dynamics [23,24,25]. The maternally inherited and slowly evolving cpDNA typically preserves lineage information from before the introduction, allowing for the identification of distinct introduction sources through haplotype differentiation [26,27]. In contrast, biparentally inherited nuclear markers (such as single nucleotide polymorphisms or microsatellites) reflect post-introduction evolutionary processes [28]. These markers reveal patterns of introgression (mainly shaped by interspecific hybridization or inter-lineage hybridization), recombination (mainly resulting from inter-population crosses within a lineage), and potential selection signatures (e.g., reduced genotype frequencies) within the introduced range [29,30]. This dual approach allows differentiation between historical admixture events that occurred near the time of introduction and more recent gene flow or hybridization dynamics during establishment and spread [31,32,33].

To address these knowledge gaps, we use *Amaranthus palmeri* S. Watson as a model system. This globally problematic dioecious weed has rapidly expanded, including a widespread distribution in China due to international soybean trade [34,35]. *A. palmeri* exhibits clear evidence of multiple introductions from mixed sources in North and South America (Figure S1), making it ideal for investigating stage-dependent changes in genetic admixture.

By conducting comparative genetic analyses on populations representing casual, naturalized, invasive, and dispersal stages within China, we aim to: (1) quantify the dynamics of genetic admixture across the invasion continuum; (2) determine the role of admixture in contributing to *A. palmeri*’s the invasion success. Our findings will advance the theoretical understanding of invasion genetics, particularly the role of genetic admixture. Additionally, they will provide crucial information for managing this herbicide-resistant agricultural “super weed” [36,37]. The analytical framework employed here provides a broadly applicable approach for understanding how genetic admixture shapes invasion trajectories across diverse taxa and ecosystems.

## 2. Results

### 2.1. Genetic Lineages and Pre-Introduction Source of cpDNA Haplotypes

**Figure 1:**
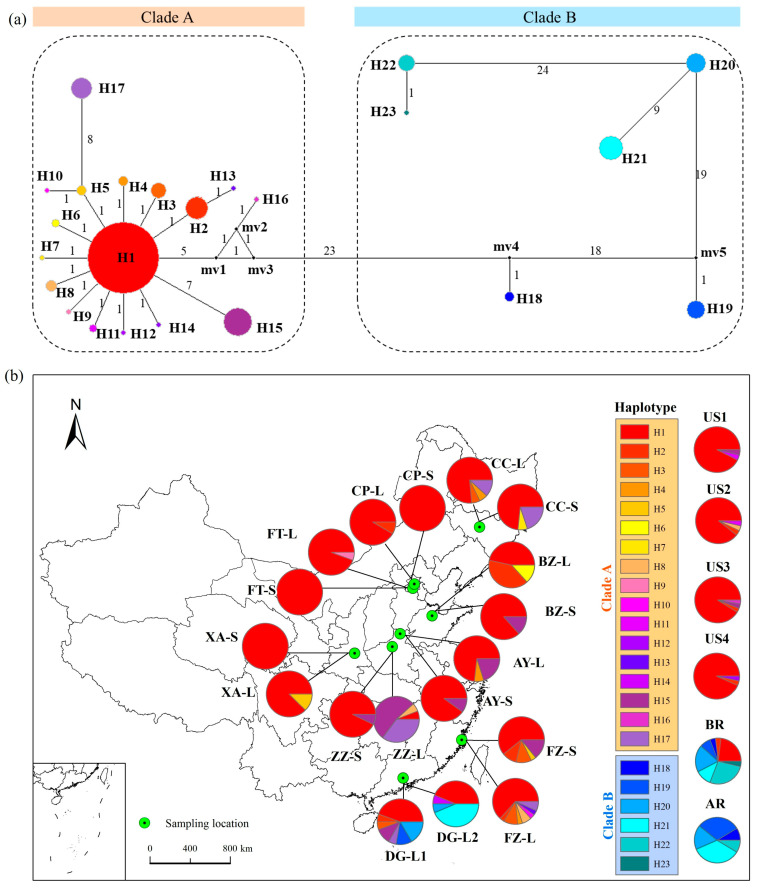
Chloroplast DNA haplotype network (**a**) and geographical distribution (**b**) of 23 haplotypes (H1–H23) in 18 introduced *Amaranthus palmeri* populations in China and source populations in the United States, Brazil, and Argentina. (**a**) The circles with different colors represent different haplotypes. The size of each circle is approximately proportional to the number of samples (*n*) harboring a haplotype, with the smallest circles representing *n* = 1 and the largest representing *n* = 359. The solid black dots (mv1 to mv5) represent missing haplotypes. Numbers next to lines represent the number of mutation steps between two connected haplotypes. Different colors represent different haplotypes; the orange represents Clade A, and the blue represents Clade B. (**b**) The circles on the map of China represent 18 seasonal introduced populations in nine geographical locations, which include different haplotypes from H1 to H23. The haplotypes contained in source populations are shown on the right side of the map.

To determine sources of introduced populations in China, a total of 23 haplotypes (H1–H23) were identified in all of these introduced populations and their source populations using the cpDNA primer with 3317 to 3401 base pairs. Phylogenetic analysis supported two genetic lineages, denoted as Clade A and Clade B (Figure 1a). Clade A included 17 haplotypes (H1 to H17) and Clade B comprised 6 haplotypes (H18 to H23). The haplotype of Clade B is at least 23 mutational steps from the haplotype of Clade A, which indicates that the two clades represent two distinct ancestral sources (Figure 1a). The most common haplotype, H1, was located in the center of the network, and radially connected to other haplotypes (Figure 1a). Haplotype H1 of Clade A had the closest relationship with the other haplotypes, meaning it is a possible ancestral haplotype for *A. palmeri*. The haplotypes in Clade B were in series with each other.

The American populations (US1, US2, US3, and US4) contained seven haplotypes (H1, H2, H8, H11, H12, H15, and H16) that belong to Clade A (Figure 1b); these haplotypes varied among the populations. The Argentine population (AR) included five haplotypes (H18, H19, H20, H21, and H22) belonging to Clade B (Figure 1b). The Brazilian population (BR) included eight haplotypes (H1, H2, H18, H19, H20, H21, H22, and H23) belonging to Clade A and Clade B (Figure 1b).

The 18 seasonal introduced *A. palmeri* populations at nine geographical locations exhibited 17 haplotypes and consisted of two distinct lineages (Figure 1b). Two casual populations (DG-L1 and DG-L2) had admixed lineages with Clade A and Clade B, which possessed four to seven haplotypes. The other four naturalized, six invasive, and six dispersal populations had a single genealogy, Clade A (Figure 1b). Four naturalized populations possessed three to eight different haplotypes, six invasive populations possessed two to four different haplotypes, and six dispersal populations possessed one to two different haplotypes. Although each of the introduced *A. palmeri* populations possessed haplotype H1 of Clade A, haplotype differentiation was present among these populations. The naturalized, invasive, and dispersal populations with leaves (-L) collected in summer had more haplotypes than the populations with seeds (-S) collected in autumn at the same location (Figure 1b). The casual population with leaves (-L1) collected in summer had different kinds and quantities of haplotypes compared to the population with leaves (-L2) in autumn at DG location (Figure 1b).

### 2.2. Post-Introduction Population Structure and Genetic Differentiation

Population differentiation index, principal component analysis, phylogenetic tree, and genetic structure were used to further determine population structure and genetic differentiation of introduced (casual, naturalized, invasive, and dispersal) populations, compared with source populations.

Population differentiation index, F_ST_ values, among all 18 introduced *A. palmeri* populations (two casual, four naturalized, six invasive, and six dispersal) and three source populations varied from 0.00 to 0.25 (Table S1). Population differentiation was higher among US (US1, US2, US3, and US4), AR, and BR populations, as indicated by higher F_ST_ values (F_ST_ = 0.09–0.17). Very little population differentiation was detected between US1, US2, US3, and US4 populations (F_ST_ = 0.00–0.03). Casual DG-L1 population had little differentiation from all source populations (F_ST_ = 0.05–0.10), and DG-L2 population had lower differentiation from AR and BR populations (F_ST_ = 0.08–0.10) than from US populations (F_ST_ = 0.13–0.16). Naturalized, invasive, and dispersal populations had lower differentiation from US populations (F_ST_ = 0.01–0.16) than from AR and BR populations (F_ST_ = 0.07–0.25) (Table S1).

PCoA showed that the AR, BR, and two casual populations (DG-L1, and DG-L2) in China belonged to Group B, and were clearly separated from the US, as well as all naturalized, invasive, and dispersal populations in China (Group A). The first principal coordinates explained 29.30% of the genetic variation (Figure S2a). The results from PCoA were basically consistent with those from the UPGMA clustering analysis, excepting for DG-L2 population was clustered to Group A (Figure S2b). Further, through principal component analysis among individuals (PCA), it was found that the individuals of the DG-L2 population were distributed in both clusters (Figure S3).

**Figure 2:**
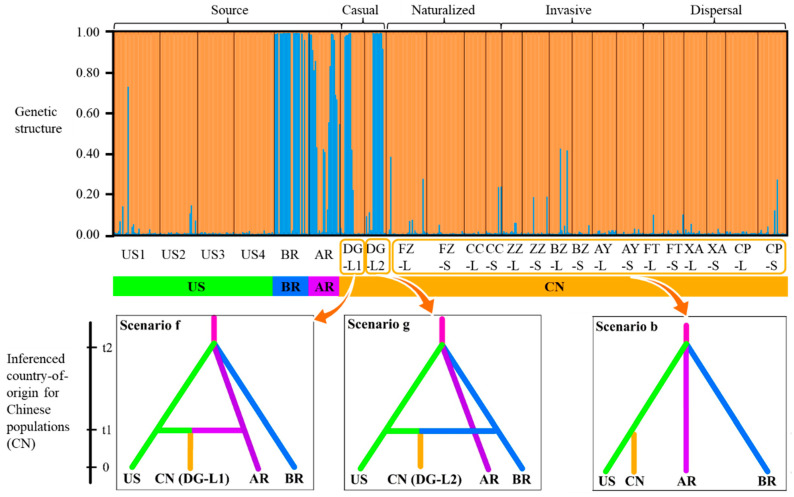
Genetic structure and inferenced country-of-origin for introduced *Amaranthus palmeri* populations in China from source populations. The figure above shows two genetic clusters of introduced (casual, naturalized, invasive, and dispersal) and source *A. palmeri* populations based on the delta K value (K = 2) by microsatellite markers. Each color (Orange or blue) represents a different genetic structure. Each individual is represented by a vertical colored (Orange or blue) line, and each population is separated by vertical black lines. The figure below shows three best scenarios (f, g, and b) of inferenced country-of-origin for introduced populations in China (CN) from the United States (US), Brazil (BR), and Argentina (AR), respectively, based on both microsatellite and cpDNA markers.

Genetic structure analysis revealed two genetic clusters based on the delta K value, which reached a maximum value when K = 2 (Figure S4a), by comparing with K = 3 and K = 4 (Figure S4b). The US populations were assigned to the orange cluster. The BR and AR populations were assigned to both blue cluster and orange cluster, while they were mainly assigned to the blue cluster (Figure 2). Two casual populations in China (DG-L1 and DG-L2) showed high levels of SSR genotypes mixture with both clusters, while four naturalized, six invasive, and six dispersal populations in China were assigned to the orange cluster (Figure 2).

### 2.3. Inferenced Country-of-Origin and Introduction Histories

The country-of-origin inference of 18 introduced *A. palmeri* populations showed that the best scenarios selected from 16 competing introduction scenarios were selected by ABC-RF simulations (Figure S5). Model selection indicated that the naturalized, invasive, and dispersal *A. palmeri* populations were consistent with the b scenario, indicating that they originated from the country of US. Two casual populations (DG-L1 and DG-L2) originated from mixed lineages. Specifically, DG-L1 originated from the countries of US and AR with admixture of divergent lineages (scenario f). DG-L2 originated from the countries of US and BR with admixture of divergent lineages (scenario g) (Table S2; Figure 2).

All 18 introduced *A. palmeri* populations in China, including invasive and naturalized populations from United States only, did not undergo reduction in effective population size (N4 > N4b) (Table S2).

### 2.4. Genetic Diversity and Hybridization Proportion

**Figure 3:**
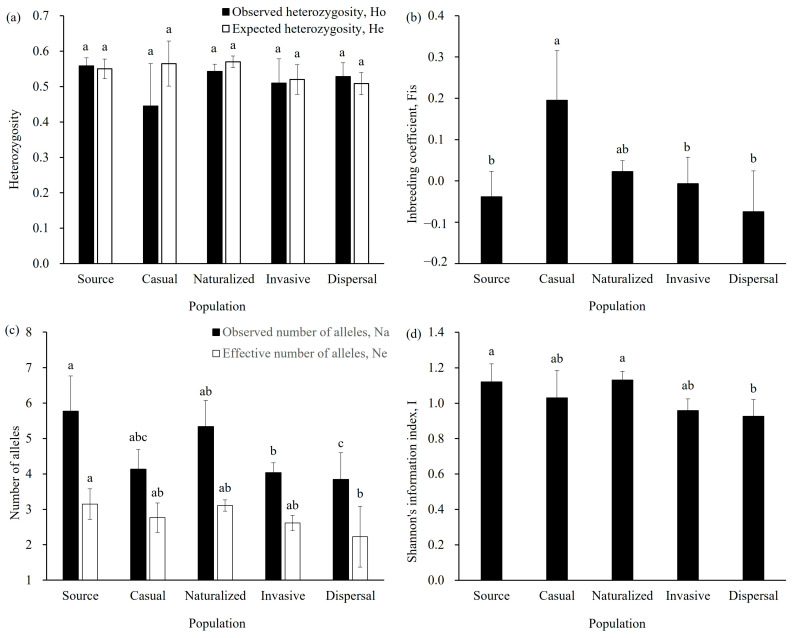
Comparison of genetic diversity parameters with heterozygosity (**a**), inbreeding coefficients (**b**), and number of alleles (**c**), as well as Shannon’s information index (**d**) for source, casual, naturalized, invasive, and dispersal *Amaranthus palmeri* populations based on microsatellite data. Heterozygosity includes observed heterozygosity (Ho) and expected heterozygosity (He). Number of alleles include both observed number of alleles (Na) and effective number of alleles (Ne). Bars labeled with different lowercase letters indicate statistically significant differences among groups as determined by one-way ANOVA followed by Tukey’s HSD test (*p* < 0.05). Groups that share a common letter are not significantly different from each other.

Genetic diversity analyses revealed consistently high levels of genetic diversity across all population categories, with comparable expected heterozygosity (He) values: source populations (He = 0.55 ± 0.03), casual (He = 0.57 ± 0.06), naturalized (He = 0.57 ± 0.02), invasive (He = 0.52 ± 0.04), and dispersal populations (He = 0.51 ± 0.03) (Figure 3a). The closely matched observed heterozygosity (Ho) and He values across these populations suggest an absence of inbreeding depression (Figure 3a). Notably, Fis values progressively decreased from casual (Fis = 0.20 ± 0.12) to naturalized (Fis = 0.02 ± 0.03), invasive (Fis = −0.01 ± 0.06), and dispersal populations (Fis = −0.08 ± 0.10) in China, reflecting a gradual reduction in inbreeding probability during the invasion process (Figure 3b). All populations showed a higher observed number of alleles (Na = 3.84–5.77) than effective alleles (Ne = 2.23–3.15), implying that selection pressures during invasion filtered out genotypes introduced through admixture (Figure 3c). This pattern aligns with the progressive decline in Shannon’s information index (I) across established populations, from naturalized (I = 1.13 ± 0.05) to invasive (I = 0.96 ± 0.07), and dispersal (I = 0.93 ± 0.10) (Figure 3d).

**Figure 4:**
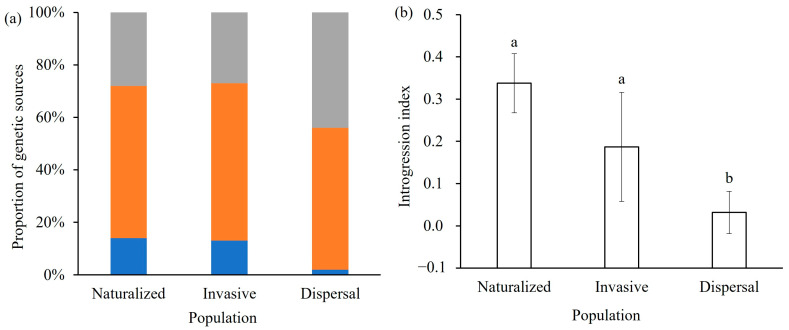
Parentage analyses (**a**) and genetic introgression index between North and South American lineages (**b**) of introduced *Amaranthus palmeri* populations based on microsatellite data. (**a**) The proportion of number of individuals with inter-lineage hybridization (blue color), inter-population crosses within North American lineage (orange color), and outcrossing within a single source population (grey color) were varied among these populations. (**b**) The changes in proportion of introgression index were decreased from naturalized, to invasive, and dispersal populations. Bars labeled with different lowercase letters indicate statistically significant differences among groups as determined by one-way ANOVA followed by Tukey’s HSD test (*p* < 0.05). Groups that share a common letter are not significantly different from each other.

Parentage analysis indicated that the proportion of number of individuals with inter-lineage hybridization decreased from naturalized (*P*_inter-lineage hybridization_ = 14 ± 11%), to invasive (*P*_inter-lineage hybridization_ = 13 ± 7%), and dispersal populations (*P*_inter-lineage hybridization_ = 2 ± 4%) (Figure 4a). More than 70% of the genetic component of all populations comes from North American lineage (*a* = 72–100%; Table S3). The introgression index of these populations from South American lineage also decreased from naturalized (34 ± 7%), to invasive (19 ± 13%), and dispersal populations (3 ± 5%) (Figure 4b). However, the high-proportion of number of individuals with inter-population crosses within North American lineage was detected in naturalized (*P*_inter-population crosses_ = 58 ± 9%), invasive (*P*_inter-population crosses_ = 60 ± 15%), and dispersal (*P*_inter-population crosses_ = 54 ± 16%) populations (Figure 4a).

### 2.5. Spatial-Temporal Dynamics with Bottlenecks and Expansions

Demographic bottleneck analysis revealed limited recent population size reductions across successfully established populations. Only two invasive (BZ-S, AY-S) and three dispersal (XA-S, CP-S, CP-L) populations exhibited genetic signatures of recent bottlenecks, characterized by significant heterozygosity excess under both the Stepwise Mutation Model (SMM) and Two-Phase Model (TPM) (*p* < 0.05) or allele frequency distribution shifts via mode-shift analysis (Table 1). In contrast, most naturalized, invasive, and dispersal populations demonstrated demographic stability with a normal L-shaped distribution of allele frequency. Notably, two populations (FZ-L, ZZ-L) showed signatures of expansion under the SMM, with significant heterozygosity deficiency (*p* < 0.05) indicative of post-bottleneck recovery (Table 1).

## 3. Discussion

Globalization has facilitated the introduction of the invasive plant *A. palmeri* to China from both North and South America [35,38]. In this study, we examined changes in the genetic admixture of mixed *A. palmeri* populations introduced to China, spanning their initial introduction, establishment, and subsequent spread. Our findings suggest that the role of genetic admixture from divergent lineages may have been overestimated.

### 3.1. North American Lineage Solely Drives Invasion Success

Our results clearly demonstrate that the naturalized, invasive, and dispersal populations in China group genetically with North American source populations (Figure 2). Notably, haplotypes of South American origin were absent from these established populations (Figure 1). Theoretically, introductions from both continents could create admixed populations containing both North and South American haplotypes, potentially enhancing invasiveness, which is a common hypothesis suggesting genetic admixture boosts invasion potential [4,39]. However, our findings contradict this expectation in this specific case. We observed that only populations derived from the North American lineage have successfully naturalized and become invasive in China (Figure 1), revealing a strong genetic bias in invasion success.

We propose that the lack of established South American haplotypes points towards strong environmental filtering. Consistent with processes involving selection or drift, our genetic diversity analyses showed that all studied populations had a higher number of observed alleles (Na = 3.84–5.77) compared to effective alleles (Ne = 2.23–3.15) (Figure 3c). Furthermore, genetic diversity, measured by Shannon’s information index, decreased progressively from naturalized (I = 1.13 ± 0.05) to invasive (I = 0.96 ± 0.07) and dispersal stages (I = 0.93 ± 0.10) (Figure 3d). We speculate that individuals carrying South American haplotypes face higher mortality rates in the introduced range, leading to the filtering out of these genotypes. This interpretation is supported by our observation of low seed germination and survival rates in South American source populations. Conversely, this filtering process likely favors North American haplotypes, perhaps due to pre-adapted traits conferring advantages in China’s environments, such as herbicide resistance or suitable flowering times [37,40].

In our study, South American haplotypes were detected only in casual populations found at the DG locations (Figure 1). Introductions from both North and South America, meaning that different individuals with genetic material from both regions were co-introduced (Figure S2). At these sites with established populations, individuals from both North and South American lineages were initially introduced and grew, but they appeared to have difficulty reproducing successfully. Therefore, the individuals with South American haplotypes likely represent only the initial introductions rather than established offspring. Their absence in established (naturalized, invasive, dispersal) populations could be attributed to competitive exclusion or dilution; the high survival rates and prolific seed production of the better-adapted North American individuals allow them to rapidly dominate the populations.

Consequently, our results suggest that the importance of genetic admixture between divergent lineages for invasion success might be context-dependent and potentially overstated in some cases [41]. This observed lineage-specific success is analogous to findings in other invasive plants, such as *Ambrosia artemisiifolia*, where introduced populations often retain adaptive traits originating from specific source regions, facilitating their establishment [42].

### 3.2. Declining South American Genetic Introgression During Invasion

However, *A. palmeri* is dioecious, facilitating cross-pollination [43]. Although plants of the South American lineage failed to establish persistent populations, their initial presence alongside North American plants (Figure 1) created opportunities for hybridization. When populations from divergent lineages co-occur, genetic admixture and subsequent introgression (the incorporation of alleles from one lineage into another via backcrossing) can be expected [44,45]. Indeed, our nuclear genetic analyses detected evidence of limited genetic introgression from the South American lineage into the established North American populations (Figure 4b). Given that only the North American maternal lineage persisted (Figure 1), this introgression likely resulted primarily from South American pollen fertilizing ovules on North American plants. Early pollen flow could thus have transferred South American alleles into the establishing North American gene pool. Genetic introgression typically results from interspecific or intra-specific (inter-lineage) hybridization [29]. As previous studies indicate that *A. palmeri* does not readily hybridize with other *Amaranthus* species [46], the observed introgression most likely stems from inter-lineage hybridization within *A. palmeri*.

Consistent with this, our admixture analyses estimated the average proportion of South American nuclear ancestry decreased across invasion stages: 14 ± 11% in naturalized, 13 ± 7% in invasive, and only 2 ± 4% in dispersal populations (Figure 4a). This declining trend strongly suggests that while inter-lineage hybridization occurred, the resulting admixture was likely disadvantageous and selected against as the invasion progressed [47,48]. While admixture can sometimes lead to hybrid vigor (heterosis), mixing divergent genetic backgrounds can also result in outbreeding depression, producing genotypes with reduced fitness [39,49,50]. Furthermore, genetic structure analysis indicates that the introduced populations in China have basically no genetic information from the South American lineage (Figure S4b). We speculate that outbreeding depression between the North and South American lineages contributed to both the failure of the South American lineage to establish and the observed decline in genetic introgression. Our preliminary crossing experiments indicating low seed set from South American female × North American male crosses support this hypothesis and would hinder population establishment from this cross direction, explaining the lack of persistent South American maternal lineages (Figure 1). This aligns with findings in model systems like *Arabidopsis thaliana*, where hybridization between divergent lines can reduce hybrid fitness [49,51].

Conversely, the persistence of North American maternal lineages (Figure 1) alongside nuclear introgression from South America (Figure 4b) is consistent with the hypothesis that the reciprocal cross—North American females pollinated by South American males—produces more viable offspring. This suggests asymmetric hybridization barriers or fitness consequences: South American pollen could successfully fertilize North American ovules (leading to the observed nuclear introgression), but the reciprocal cross likely produced nonviable or less fit offspring. Such directional compatibility could explain both the limited, declining nuclear introgression and the complete absence of South American maternal lineages in established populations.

Therefore, despite the theoretical potential for hybridization to generate novel, advantageous genotypes, the steady decline of South American alleles indicates that admixture between the North and South American lineages did not play a substantial positive role, and may even have been detrimental, to *A. palmeri*’s invasion success in China. These results also highlight the value of studying failed or partially successful introductions involving hybridization between distinct lineages to gain a more complete understanding of invasion mechanisms [52]. Future research should directly assess the fitness of hybrid genotypes resulting from reciprocal crosses. Our findings emphasize that, in this invasion, pre-existing lineage-specific adaptations appear more critical for success than any potential benefits (or despite potential drawbacks) of inter-lineage hybrid vigor [53]. Importantly, however, this conclusion applies specifically to inter-lineage admixture; gene exchange within the successful North American lineage may tell a different story.

### 3.3. Inter-Population Crosses Within North American Lineage Facilitates Invasion

In contrast to the unwanted effects of inter-lineage hybridization, our results indicate that inter-population crosses within the North American lineage appear crucial for invasion success. We detected high levels of inter-population crosses derived from different North American source populations within the naturalized, invasive, and dispersal populations (Figure 4a). Furthermore, decreasing inbreeding coefficients (Fis) from casual (Fis = 0.11–0.28) to naturalized (Fis = 0.01–0.06), invasive (Fis = −0.05–0.09), and dispersal (Fis = −0.21–0.08) populations strongly suggest frequent inter-population crosses occurred throughout the invasion process (Figure 3b).

This pattern likely results from two interconnected processes. Firstly, multiple introductions from diverse North American sources probably acted as a form of genetic rescue. This maintained high genetic diversity (heterozygosity, He = 0.44–0.59; Figure 3a), sometimes even higher than in the source populations. Multiple introductions, even from the same broad lineage, bring diverse genotypes, increasing the raw material available for natural selection [42]. Merging gene pools from multiple sources boosts standing genetic variation [8,54], which can preserve pre-adapted genotypes or generate novel, potentially advantageous trait combinations through recombination [10,55]. Subsequently, adaptive filtering likely occurred: natural selection presumably favored locally adapted genotypes, leading to a gradual reduction in overall diversity from the naturalized (He = 0.55–0.59) to the invasive (He = 0.44–0.55) and dispersal (He = 0.48–0.54) stages (Figure 3a). This reduction suggests that only the most suitable genotypes persisted and spread in the new environments [54,56], while a larger initial pool of genotypes increased the probability that such suitable genotypes were present [57].

Secondly, ongoing inter-population crosses within the North American lineage helped populations avoid severe genetic bottlenecks associated with introduction (Table 1 and Table S2). Crosses between different introduced populations generate further genetic variation through recombination [58,59,60]. Our data show that a substantial proportion of individuals (averaging 58% in naturalized, 60% in invasive, 54% in dispersal populations) resulted from gene exchange among different North American source populations (Figure 4a). This extensive gene exchanges likely facilitated establishment and expansion by mitigating the effects of genetic bottlenecks [61,62]. Indeed, our successfully established *A. palmeri* populations did not exhibit a reduction in long-term effective population size (N4 > N4b) (Table S2) and largely avoided significant genetic bottlenecks (Table 1), although some bottleneck signals detected in later (invasive/dispersal) stages might reflect recent founding events during range expansion [63]. In the long term, these inter-population crosses may promote rapid evolution and local adaptation [64,65], contributing significantly to the invasion success of these introduced populations [66]. This mirrors findings in other invasive species like *Solidago altissima*, where multiple introductions facilitated rapid adaptation [67].

These findings support the hypothesis that multiple introductions from within a single successful lineage can promote invasion through genetic enrichment and adaptive flexibility [24,68]. This challenges the assumption that genetic admixture between distinctly different exotic lineages is always a prerequisite for invasion success. While our study successfully employed cost-effective methods to track genetic admixture dynamics across invasion stages, the relatively low resolution of these approaches limits fine-scale insights into adaptive genotypes. Future work leveraging high-throughput genomic sequencing could address this gap by pinpointing specific admixed loci linked to fitness advantages and testing whether intra-lineage crosses—a key driver of *A. palmeri*’s invasion—represents a broader strategy among successful invasive taxa.

## 4. Materials and Methods

### 4.1. Study Species and Sample Collection

Since from 1985, seeds of *Amaranthus palmeri* S. Watson have been frequently introduced as contaminants in soybean imports to provinces in southern, central, northern, and northeastern China [34,35]. Our multi-year field investigations and analyses of genetically modified (GM) soybean importation trade data indicate that *A. palmeri* is primarily distributed near grain importation facilities. These localities have documented imports from the United States, Argentina, and Brazil, with shipments occurring more than twice annually per country since the late 1990s. In some areas, *A. palmeri* has spread beyond its initial introduction sites (e.g., grain importation factories). Consequently, populations exhibit distinct characteristics based on their establishment status (transient or self-sustaining) and spread performance (distribution range), enabling classification as casual, naturalized, invasive, and dispersal [18,19,21].

For this study, we selected nine representative geographical populations of *A. palmeri* across China. Specifically, the Dongguan (DG) population was classified as casual, while Fuzhou (FZ) and Changchun (CC) were categorized as naturalized. Populations in Anyang (AY), Zhengzhou (ZZ), and Binzhou (BZ) were identified as invasive, and those in Xi’an (XA), Fengtai (FT), and Changping (CP) were designated as dispersal (Table S4). There populations originated from introductions in the United States, Brazil, and Argentina. We collected seeds from four populations in the United States (designated US1, US2, US3, and US4), one population from Brazil (BR), and one population from Argentina (AR). These United States (US1-4), Brazil (BR) and Argentine (AR) populations were recognized as source populations for subsequent introductions.

Because new seeds are continuously introduced through trade, individuals within populations may originate from two sources: newly introduced seeds or offspring produced by established plants. Considering compensatory gene flow, demography disturbance in introduced regions, genetic composition of a population should be varied between the summer vegetative period and autumn reproductive period. To account for seasonal variation caused by ongoing introductions, environmental selection, stochasticity, or disturbance, populations were sampled in both summer (vegetative phase) and autumn (reproductive phase). Summer samples were designated leaf seasonal populations (e.g., XA-L), with leaves collected for genotyping; while autumn samples were designated seed seasonal populations (e.g., XA-S), with seeds collected for analysis (Table 2). Among them, DG population is vegetative growth periods in both spring and autumn, so we collected leaves (DG-L1 and DG-L2), respectively. All materials were gathered between 2019 and 2021 at the nine study sites. Individuals were sampled at intervals of 5–50 m, adjusted to local population density (Table 2).

### 4.2. DNA Extraction and Molecular Identification

All collected seeds were stored in paper envelopes and grown in an isolation greenhouse. At the four-leaf stage, one or two young, healthy leaves of each seedling and collected leaves were harvested for total genomic DNA extraction using the Hi-Fast Plant Genomic DNA Kit (GeneBetter Biotech, Beijing, China). Molecular genotyping identification of all collected samples from 18 introduced and three source *A. palmeri* populations was conducted by PCR amplification using the internal transcribed spacer (ITS) region with the primer pair ITS1 and ITS4 [69]. After searching and comparing the amplified sequences of all collected samples with the online Taxonomy Browser of the National Center for Biotechnology Information (NCBI), nuclear gene sequences of 499 individuals were identified by BLAST (https://blast.ncbi.nlm.nih.gov/Blast.cgi, accessed on 20 May 2024) and used in the following experiments.

### 4.3. Sequencing and Data Analysis

#### 4.3.1. Genetic Lineages and Pre-Introduction Source Analysis

To detect genetic lineages and geographical distribution of cpDNA haplotypes, three primer pairs (petB-petD, ropB-trnC, and petA-petJ) targeting intergenic regions were selected from among the 21 universal primers reported by Dong et al. (2012) and used for PCR amplification of 499 samples from the 18 introduced and three source *A. palmeri* populations (Table S5) [70]. The PCR products were evaluated on a 1% agarose gel and sequenced from both the 3′ and 5′ ends by Sangon Biotech Services Company Ltd. (Beijing, China). After sequencing, raw sequence data were processed using BIOEDIT software (version 7.0.9) (Scotts Valley, CA, USA) [71], and sequences of each sample with 3 cpDNA primer pair were manually edited and assembled into a contig. Sequences of the three cpDNA regions were combined and saved in FASTA format. cpDNA haplotypes were inferred from aligned cpDNA sequences using MEGA (version 7) (Mega Limited, Auckland, New Zealand) and DnaSP (version 5.10.01) (Universität de Barcelona, Barcelona, Spain) [72].

A genealogical haplotype network based on the median-joining method was then constructed using Network (version 4.6.13) (Microsoft, Washington, DC, USA). The genealogical haplotype network was used to estimate the degree of relatedness per haplotype, and indels were treated as single mutational events [73]. Possible genetic lineages of all detected haplotypes were determined through haplotype relationships using the genealogical haplotype network. The haplotype distribution in three source (US, BR, and AR) and 18 seasonal introduced (two casual, four naturalized, six invasive, and six dispersal) populations in nine geographical locations was visualized using ArcMap (version 10.2) (Environmental Systems Research Institute, Redlands, CA, USA).

#### 4.3.2. Post-Introduction Population Structure and Genetic Differentiation Analysis

To further detect the genetic structure and population differentiation of casual, naturalized, invasive, and dispersal populations in China, compared with source populations in United States, Argentina, and Brazil, all 499 genomic DNA samples were genotyped using 12 microsatellites primers (Table S6). Six of these primers were designed using SSRHunter (version 1.3) (Nanjing Agricultural University, Nanjing, China) and Primer 3.0 (version 5) (Premier, Vancouver, BC, Canada) based on genomic data of *A. palmeri* [74]. These primers were labeled with 5-carboxyfluorescein (5-FAM). The other six primers were from Lee et al. (2008) and were labeled with 5-carboxy-X-rhodamine (5-ROX) [75]. The PCR reaction volumes and cycling conditions were the same as those described above for the cpDNA assay (Table S5). PCR products were evaluated on a 1% agarose gel and sent to Sangon Biotech Services Company Ltd. for genotyping with an internal size standard (GeneScan^TM^ 500 LIZ, Applied Biosystems, Waltham, MA, USA). The sizes of the amplified bands (i.e., alleles) were scored using GeneMapper (version 3.2) (Applied Biosystems, Waltham, MA, USA). All allelic data were converted to the format required by GenAlEx 6.5 software (Australian National University, Canberra, Australia) [76] for further genetic analysis.

We analyzed population differentiation using four complementary approaches. Firstly, we calculated population differentiation index (i.e., pairwise fixation index, F_ST_ values) based on genetic distance using Arlequin (version 3.5.2.2) (University of Berne, Baltzer Strasse, Berne, Switzerland) [77]. Higher values indicate larger differentiation among populations. Secondly, we employed principal coordinate analysis (PCoA) using GenAlEx (version 6.5) (Rutgers University, New Brunswick, NJ, USA) [76]. We also conducted principal component analysis (PCA) among individuals to better capture genetic variation within and across populations using GenAlEx (version 6.5) (Rutgers University, New Brunswick, NJ, USA) [76]. Thirdly, the unweighted pair group method with arithmetic mean (UPGMA) clustering was used to analyze population differentiation by the software of POPTREE (version 2) (Kagawa University, Takamatsu, Japan). Finally, the genetic structure of each population was analyzed to determine ancestor sources using STRUCTURE (version 2.3.4) (Stanford University, CA, USA) [78]. Analyses were performed according to an admixture ancestor model with associated allele frequencies. K clusters were estimated by simulating K values ranging from 1 to 24. A burn-in and run length of the Monte Carlo Markov Chain (MCMC) of 50,000 and 100,000 permutations, respectively, with 10 runs per K-value were performed. The best K-value was estimated according to the K method [79] using Structure Harvester (version 6.193) (Santa Cruz, CA, USA) [80].

#### 4.3.3. Inferring Country-of-Origin and History of Introduced Populations

To further determine the country-of-origin of 18 introduced *A. palmeri* populations (two casual, four naturalized, six invasive, and six dispersal populations) in China and how their population sizes changed over time, we compared possible introduction scenarios using approximate Bayesian computation (ABC) analyses based on haplotype and genetic variation. A recently developed random forest (RF) machine learning tool based on ABC was used to conduct model selection and parameter estimation in DIYABC-RF (version 1.0) (Montpellier, France) [81] from a total of 16 competing introduction scenarios (Scenario a to Scenario p) (Figure S2). We then estimated variation between parameters N4 (the current effective population sizes) and N4b (the past effective population sizes) at the best scenario of each population to investigate recent changes in population size of all 18 introduced *A. palmeri* populations. An *A. palmeri* population was considered to be introduced if it had a possible effective population size decrease after invasion (i.e., N4 < N4b).

#### 4.3.4. Genetic Diversity and Hybridization Detection

To determine populations’ genetic changes after invasion, genetic diversity and inbreeding coefficients of casual, naturalized, invasive, and dispersal populations were analyzed and compared with those of source populations. This included evaluating and comparing the genetic diversity values of each *A. palmeri* population based on expected heterozygosity (He), observed heterozygosity (Ho), Shannon’s information index (I), the observed number of alleles (Na), and the effective number of alleles (Ne). High values of He and other indexes indicate high genetic diversity [82]. Furthermore, inbreeding coefficient (Fis) values were calculated to assess the deficit of heterozygotes caused by inbreeding within a population [83] using POPGENE (version 1.32) software (University of Alberta, Alberta, Canada).

To further determine genetic sources of 18 introduced *A. palmeri* populations (two casual, four naturalized, six invasive, and six dispersal populations), we conducted a parentage analysis based on the similarity of Allele. Given the dioecism of *A. palmeri*, two parentages of each individual in *A. palmeri* populations were inferred and simulation of parentage analysis based on likelihood approach by Cervus (version 3.0) software (Montana State University, Bozeman, MT, USA) [84]. Each individual in the introduced populations was assigned the most likely parent pairs [85]. We calculated that the proportion of number of individuals with inter-lineage hybridization (*P*_inter-lineage hybridization_), which means that the parents of one individual were detected by coming from different lineages. We also calculated that the proportion of number of individuals with inter-population crosses within North American lineage (*P*_inter-population crosses_), which means that the parents of one individual were detected by coming from different populations within the same lineage. The left individuals without detecting inter-lineage hybridization and inter-population crosses are thought to occur outcrossing within a single source population (*P*_outcrossing_).

To further determine the genetic contribution of hybridization between two divergent lineages, introgression index was estimated based on allele frequencies of *A. palmeri* populations using the least squares method in R 3.5.3 with the following linear regression model:

$$Y_{ij}=aX_{1ij}+bX_{2ij}+c$$

In this equation, *Y_ij_* is the *i*th allele frequency at the *j*th locus of an introduced *A. palmeri* population; *X*_1*ij*_ is the *i*th allele frequency at the *j*th locus of populations in United States; *X*_2*ij*_ is the *i*th allele frequency at the *j*th locus of populations in South America (Argentina and Brazil); *a* and *b* are regression coefficient and *c* is the intercept of estimating admixture [86]. The regression coefficient, *a*, reflects the contribution of populations in United States to a hybrid family, and *b* reflects the contribution of populations in South America to a hybrid family.

#### 4.3.5. Spatial-Temporal Dynamics of Genetic Bottleneck and Expansion Analyses

Finally, to assess spatial-temporal dynamics of naturalized, invasive, and dispersal populations in different seasons and locations, bottlenecks and expansions of post-introduction effective population size were detected using the microsatellite data. The effective population size reductions, or bottlenecks, were analyzed using heterozygosity excess measurements with the stepwise mutation model (SMM), the two-phased model of mutation (TPM), and the mode-shift test in BOTTLENECK (version 1.2.02) (Montferrier-sur-Lez Cedex, France) [87,88]. Additionally, effective population size expansions were analyzed using heterozygosity deficiency measurements with SMM and TPM using BOTTLENECK (version 1.2.02) software (Montferrier-sur-Lez Cedex, France). Significant heterozygosity excess or a mode shift in allele frequency indicates a genetic bottleneck due to population size reduction, while significant heterozygosity deficiency indicates effective population size expansion (*p* < 0.05). A normal L-shaped distribution of allele frequency indicates a stable population.

## 5. Conclusions

With the development of globalization, the notorious invasive plant, *Amaranthus palmeri*, has been introduced to large-scale regions in China through the imported soybean trade. It originated from mixed sources in both North and South America. Our research reveals that throughout the invasion process—spanning transport, introduction, establishment, and spread—only haplotypes from the North American lineage persisted. Over time, the genetic contribution from the South American lineage diminished, and the successfully established populations became dominated by the North American lineage with a low level of genetic introgression. However, a high proportion of inter-population crosses (>54%) and a low inbreeding coefficient (Fis < 0.1) suggest that multiple-source introductions of North American lineages may have played a key role in increasing genetic diversity and overcoming population bottlenecks in introduced *A. palmeri* populations in China. Our results indicate that the primary driver of the successful invasion of *A. palmeri* may be inter-population crosses within the North American lineage. In contrast, inter-lineage hybridization between North and South American lineages might not be critical for population establishment. However, whether outbreeding depression, which could arise from hybridization, occurs should be assessed in future studies [88,89]. Our findings may provide potential genetic strategies for controlling invasive *A. palmeri* populations.

## Figures and Tables

**Table 1:** Bottleneck and expansion analyses. Bottleneck and expansion analyses of source and introduced *Amaranthus palmeri* populations based on allele frequency by SMM and TPM as well as mode-shift tests.

Population	SMM			TPM			Mode Shift
	Deficiency	Excess	Deficiency or Excess	Deficiency	Excess	Deficiency or Excess	
FZ-L	0.04 *	0.97	0.08	0.21	0.80	0.43	L
FZ-S	0.27	0.75	0.54	0.85	0.16	0.33	L
CC-L	0.55	0.48	0.95	0.87	0.15	0.30	L
CC-S	0.09	0.92	0.17	0.27	0.75	0.54	L
AY-L	0.07	0.94	0.14	0.40	0.62	0.81	L
AY-S	0.92	0.09	0.17	0.99	0.02 *	0.04 *	Y
ZZ-L	0.27	0.74	0.54	0.77	0.25	0.50	L
ZZ-S	0.02 *	0.99	0.04 *	0.05	0.95	0.10	L
BZ-L	0.50	0.52	1.00	0.85	0.16	0.33	L
BZ-S	0.87	0.15	0.30	0.99	0.01 *	0.03 *	Y
XA-L	0.32	0.71	0.64	0.81	0.21	0.41	L
XA-S	0.96	0.05	0.09	1.00	0.00 *	0.01 *	Y
FT-L	0.52	0.52	1.00	0.92	0.09	0.18	L
FT-S	0.05	0.95	0.10	0.43	0.60	0.86	L
CP-L	0.98	0.03 *	0.06	0.99	0.01 *	0.03 *	L
CP-S	0.82	0.20	0.39	1.00	0.00 *	0.01 *	L

Notes: “L” indicates a normal L distribution of allele frequency. “Y” indicates the existence of a mode shift in allele frequency. * Values significant at *p* < 0.05.

**Table 2:** Information about 499 samples of *Amaranthus palmeri* collected from three sources and nine geographical populations in China.

Country	No.	Coordinates (N, E)	Location	Year	Collected Material	Code	Sample Size	Category
United States	1	-		2019	Seeds	US1	34	Source
		-		2020	Seeds	US2	28	
		-		2021	Seeds	US3	27	
		-		2022	Seeds	US4	30	
Brazil	2	-		2019–2022	Seeds	BR	26	Source
Argentina	3	-		2019–2022	Seeds	AR	23	Source
China	4	(22°59′41″, 113°32′58″)	Dongguan	2019	Leaves	DG-L1	18	Casual
		(22°59′41″, 113°32′58″)		2019	Leaves	DG-L2	16	
	5	(25°57′36″, 119°31′12″)	Fuzhou	2021	Leaves	FZ-L	30	Naturalized
		(25°57′36″, 119°31′12″)		2021	Seeds	FZ-S	28	
	6	(43°58′57″, 125°27′50″)	Changchun	2019	Leaves	CC-L	17	Naturalized
		(43°58′57″, 125°27′50″)		2019	Seeds	CC-S	15	
	7	(35°53′12″, 114°23′13″)	Anyang	2021	Leaves	AY-L	15	Invasive
		(35°53′12″, 114°23′13″)		2021	Seeds	AY-S	20	
	8	(34°48′02″, 113°24′19″)	Zhengzhou	2021	Leaves	ZZ-L	17	Invasive
		(34°48′02″, 113°24′19″)		2021	Seeds	ZZ-S	14	
	9	(37°08′38″, 118°08′42″)	Binzhou	2019	Leaves	BZ-L	15	Invasive
		(37°08′38″, 118°08′42″)		2019	Seeds	BZ-S	15	
	10	(34°25′49″, 109°17′05″)	Xi’an	2021	Leaves	XA-L	16	Dispersal
		(34°25′49″, 109°17′05″)		2021	Seeds	XA-S	12	
	11	(39°48′38″, 116°22′10″)	Fengtai	2021	Leaves	FT-L	18	Dispersal
		(39°48′38″, 116°22′10″)		2021	Seeds	FT-S	20	
	12	(39°41′33″, 116°15′32″)	Changping	2019	Leaves	CP-L	24	Dispersal
		(39°41′33″, 116°15′32″)		2019	Seeds	CP-S	21	

## Data Availability

All the data analyzed during this study are included in this published article and its Supplement Information. The sequences have been uploaded to the National Genomics Data Center of China National Center for Bioinformation (CNCB) (Accession number range from C_AA113703.1 to C_AA113725.1).

## Supplementary Materials

The supporting information can be downloaded at: https://www.mdpi.com/article/10.3390/ijms26178128/s1.
