# Supplementary material for: Dynamic Genetic Changes Reveal: Intra-Lineage Diversity, Not Admixture, Explains Amaranthus palmeri’s Success in China
Source: Int J Mol Sci. 2025 Aug 22;26(17):8128. doi: 10.3390/ijms26178128 (PMC12427824; doi:10.3390/ijms26178128)
Supplement: Supplementary file 1 [file ijms-26-08128-s001.zip › ijms-3754513-supplementary.pdf]

Supporting Information

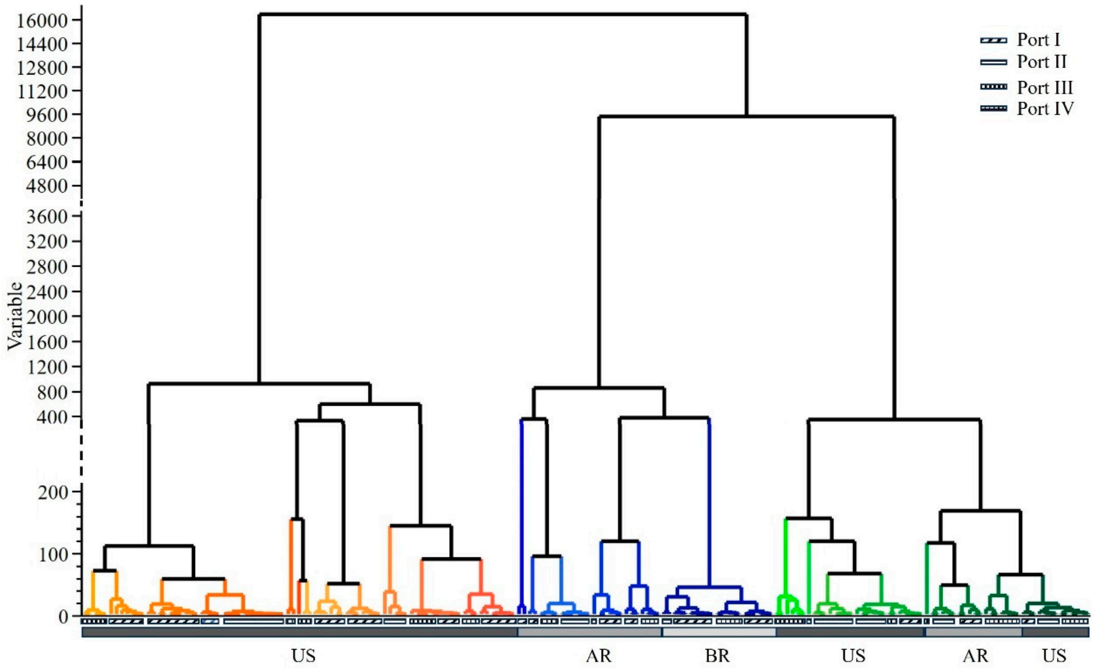

Figure S1. Multiple-origin introduction pathway for *Amaranthus palmeri* in China based on stable isotope analysis of carbon ($\delta^{13}C$) and nitrogen ($\delta^{15}N$). Propagules entered primarily through ports that serve as gateways for soybean imports originating from three source countries: the United States (US), Argentina (AR), and Brazil (BR). Four ports importing soybean from over 30 regions across these three countries were selected. The isotopic profiles indicate both genetic admixture and recurrent introduction events, demonstrating that introductions originated from multiple geographically distinct populations within these source countries.

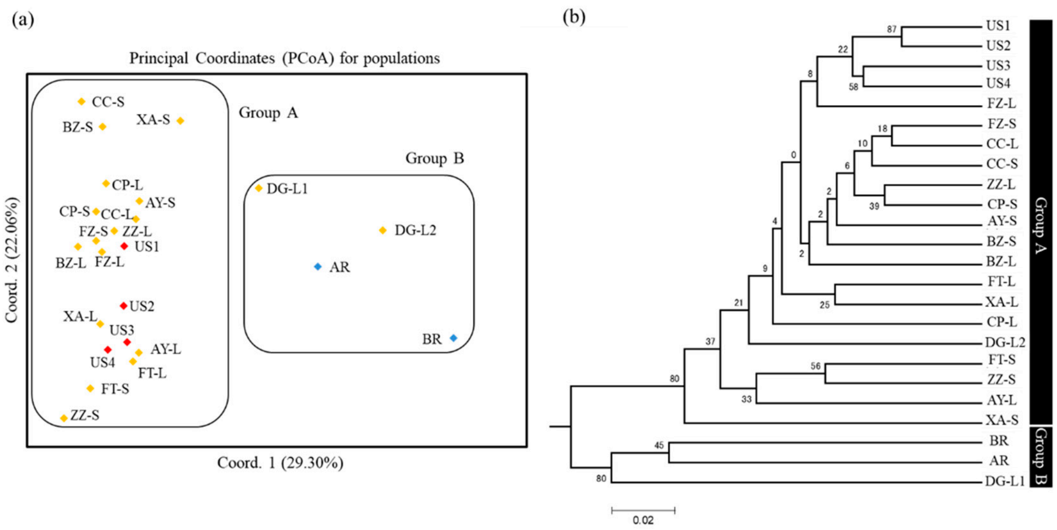

Figure S2. Principal coordinates analysis (PCoA) (a) and UPGMA dendrogram (b) of introduced and source *Amaranthus palmeri* populations based on microsatellite data. In the UPGMA dendrogram, numbers above or below the branches represent the level of statistical support of a cluster. The scale bar represents the branch length based on Nei's genetic distance.

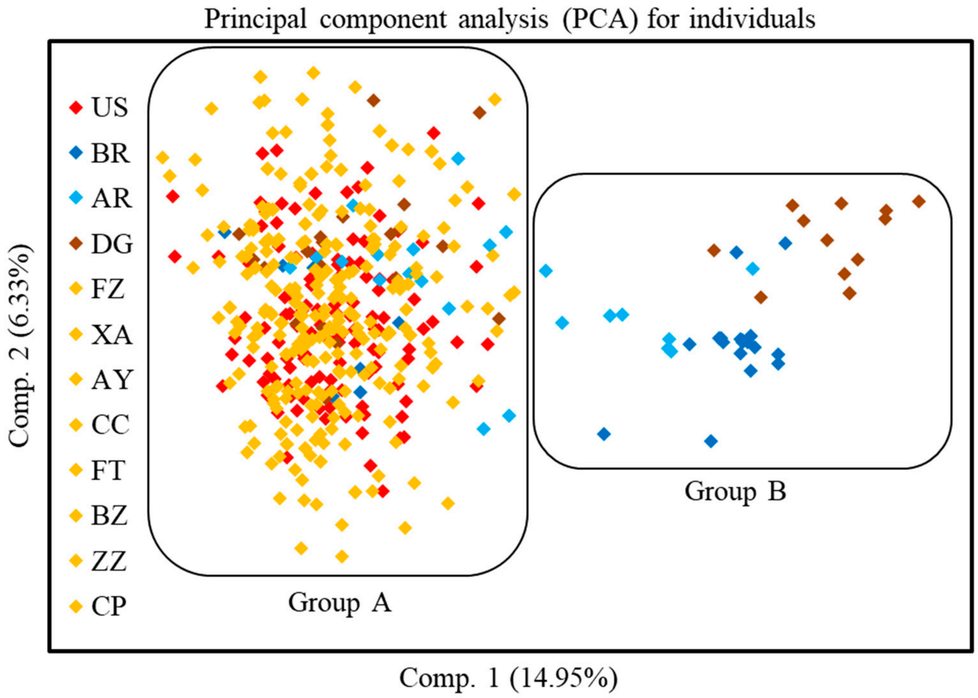

Figure S3. Principal component analysis (PCA) of individuals within introduced and source *Amaranthus palmeri* populations based on microsatellite data.

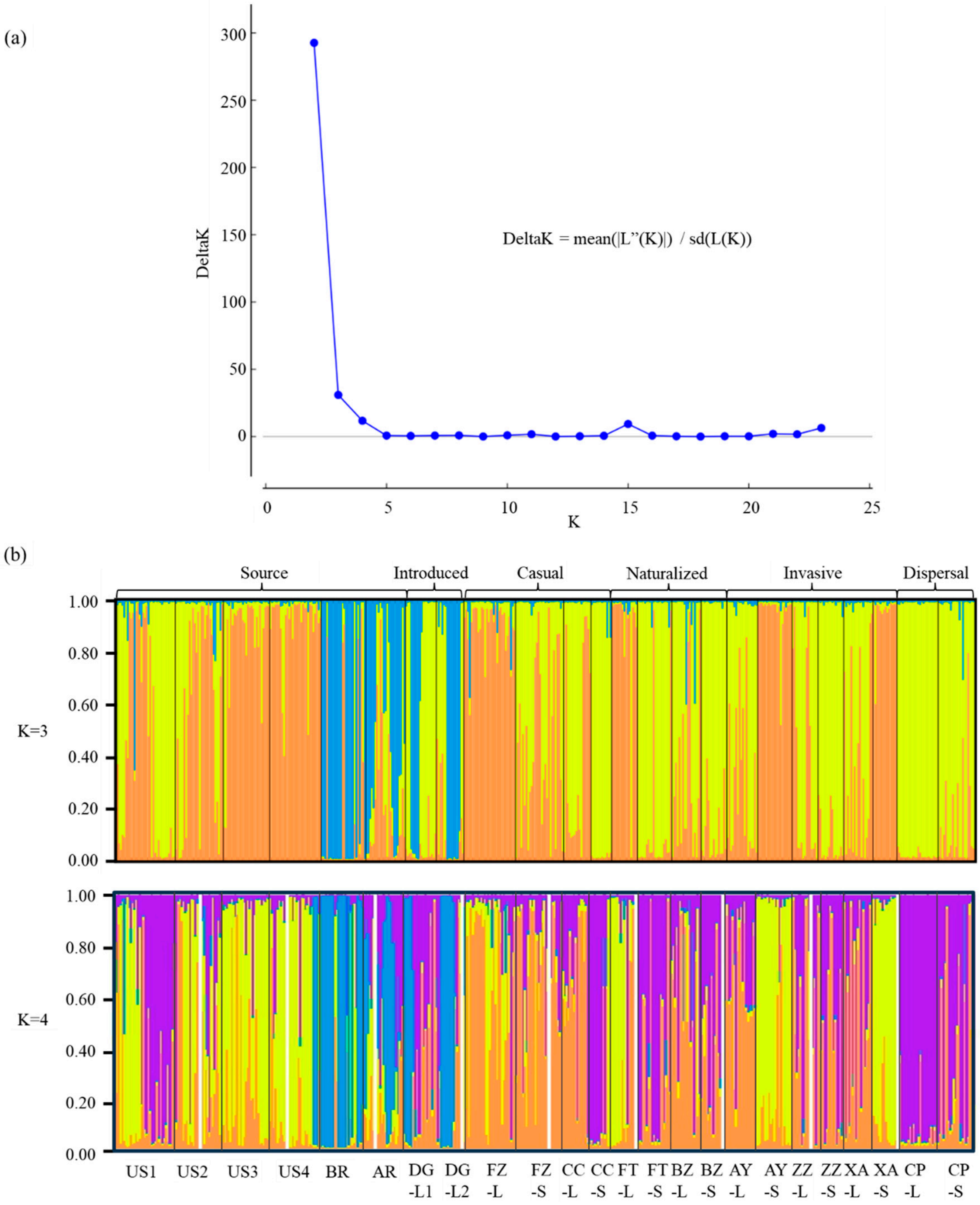

Figure S4. Relationship between the number of genetic groups (K) and the estimated delta K (△k) value (a) and genetic structure on K=3 and K=4 (b) based on the microsatellite dataset.

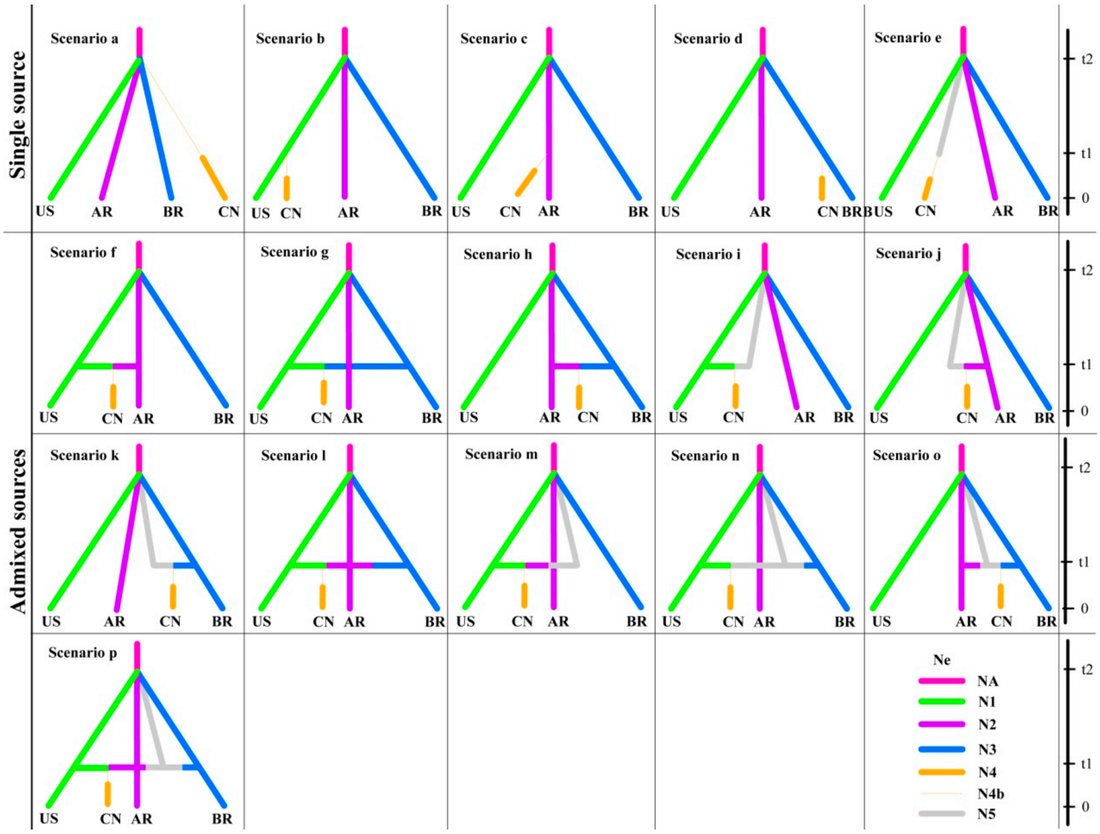

Figure S5. Competing scenarios for introduced *Amaranthus palmeri* populations. A total of 16 competing introduction scenarios (Scenario a to Scenario p) were considered by analyzing the relationships of the 18 introduced populations (two casual, four naturalized, six invasive, and six dispersal) in China with the three putative sources (US, AR, and BR) in country level. For each introduction scenario, we allowed these three sources to be linked by an overarching native population. We also considered an unsampled source ("ghost" source). The past effective population sizes of introduced *A. palmeri* populations in China were denoted as N4b. The current effective population sizes of introduced populations in China were denoted as N4. After identifying all competing introduction scenarios, we included all possible summary statistics provided by DIYABC-RF and ran 20,000 simulations for each scenario. The scenario with the highest classification vote was selected from a classification forest of 500 trees as the best scenario for each of the 18 introduced *A. palmeri* populations, and its posterior probability was calculated. Finally, we estimated parameters N4 and N4b by running 100,000 simulations for the best scenario of each population and inferring posterior distribution values of these two parameters with a classification forest of 500 trees. Variation between N4 and N4b was used to investigate recent changes in population size of all 18 introduced *A. palmeri* populations. An *A. palmeri* population was

considered to be introduced if it had a possible effective population size decrease after invasion (i.e., N4 < N4b). NA represents the effective population size of the ancestral lineage at time t2; t1 represents divergence time for the depicted event. The current effective population sizes of *A. palmeri* populations in US, AR, BR, introduced populations in China (CN), and unsampled populations ("ghost" populations) were denoted as N1, N2, N3, N4, and N5, respectively. The past effective population sizes of introduced populations in China were denoted as N4b.

Table S1 Population pairwise F_{ST} values for 18 introduced and three source populations based on microsatellite data.

Population	US1	US2	US3	US4	BR	AR	DG-L1	DG-L2	FZ-L	FZ-S	XA-L	XA-S	AY-L	AY-S	CC-L	CC-S	FT-L	FT-S	BZ-L	BZ-S	ZZ-L	ZZ-S	CP-L	CP-S
US1	0.00																							
US2	0.00	0.00																						
US3	0.03	0.01	0.00																					
US4	0.02	0.01	0.02	0.00																				
BR	0.16	0.15	0.17	0.16	0.00																			
AR	0.09	0.09	0.11	0.12	0.10	0.00																		
DG-L1	0.05	0.06	0.08	0.08	0.10	0.07	0.00																	
DG-L2	0.13	0.14	0.16	0.16	0.10	0.08	0.08	0.00																
FZ-L	0.03	0.04	0.06	0.05	0.17	0.10	0.05	0.14	0.00															
FZ-S	0.01	0.02	0.04	0.03	0.18	0.10	0.05	0.15	0.01	0.00														
XA-L	0.04	0.04	0.04	0.04	0.19	0.12	0.10	0.20	0.05	0.02	0.00													
XA-S	0.09	0.11	0.16	0.14	0.23	0.16	0.10	0.21	0.11	0.11	0.14	0.00												
AY-L	0.05	0.05	0.04	0.05	0.16	0.10	0.08	0.12	0.06	0.05	0.07	0.18	0.00											
AY-S	0.02	0.03	0.05	0.05	0.18	0.11	0.04	0.15	0.03	0.02	0.04	0.09	0.06	0.00										
CC-L	0.01	0.02	0.05	0.04	0.16	0.07	0.03	0.12	0.01	0.00	0.04	0.09	0.05	0.03	0.00									
CC-S	0.04	0.07	0.11	0.10	0.25	0.13	0.07	0.17	0.03	0.03	0.11	0.11	0.10	0.05	0.02	0.00								
FT-L	0.06	0.06	0.08	0.09	0.20	0.14	0.12	0.20	0.11	0.10	0.06	0.16	0.13	0.09	0.07	0.17	0.00							
FT-S	0.03	0.02	0.04	0.03	0.17	0.12	0.07	0.16	0.03	0.03	0.04	0.14	0.06	0.05	0.04	0.11	0.06	0.00						
BZ-L	0.02	0.03	0.05	0.05	0.20	0.13	0.06	0.18	0.04	0.02	0.05	0.14	0.09	0.05	0.03	0.05	0.09	0.05	0.00					
BZ-S	0.04	0.08	0.10	0.10	0.23	0.12	0.06	0.16	0.03	0.03	0.10	0.13	0.09	0.06	0.02	0.00	0.16	0.10	0.05	0.00				
ZZ-L	0.01	0.02	0.04	0.05	0.17	0.08	0.04	0.13	0.02	0.01	0.03	0.11	0.05	0.01	0.01	0.03	0.10	0.04	0.02	0.03	0.00			
ZZ-S	0.07	0.05	0.09	0.07	0.21	0.16	0.12	0.19	0.07	0.07	0.08	0.18	0.11	0.10	0.07	0.15	0.10	0.03	0.08	0.14	0.08	0.00		
CP-L	0.03	0.05	0.07	0.07	0.21	0.12	0.06	0.17	0.07	0.05	0.08	0.13	0.09	0.04	0.03	0.05	0.10	0.08	0.03	0.06	0.03	0.12	0.00	
CP-S	0.02	0.03	0.06	0.06	0.20	0.12	0.05	0.16	0.05	0.03	0.09	0.13	0.09	0.03	0.02	0.04	0.09	0.07	0.03	0.04	0.02	0.10	0.02	0.00

Table S2 Competing scenarios and parameter values of *Amaranthus palmeri* populations. Classification vote, posterior probability, and posterior median estimates (95% credible interval) of two parameter values (N4 and N4b) for the best scenario (scenario b, scenario f, scenario g) selected from 16 competing introduction scenarios of 18 introduced *A. palmeri* populations in China.

Population	Best scenario	Classification vote	Posterior probability	N4	N4b
DG-L1	f	49	0.49	5830 (2096-9824)	5022 (677-9417)
DG-L2	g	45	0.48	5166 (841-9491)	5067 (617-9623)
FZ-L	b	177	0.55	7355 (2672-9851)	5214 (745-9533)
FZ-S	b	135	0.54	5997 (1525-9667)	5498 (1002-9678)
CC-L	b	150	0.56	6549 (1940-9641)	5152 (1171-9474)
CC-S	b	106	0.49	6403 (1937-9692)	5502 (1185-9619)
AY-L	b	126	0.52	5692 (1459-9600)	5575 (947-9574)
AY-S	b	130	0.47	5583 (786-9791)	5356 (972-9619)
ZZ-L	b	118	0.48	6015 (1410-9665)	5910 (777-9628)
ZZ-S	b	103	0.51	5818 (1585-9571)	5163 (773-9570)
BZ-L	b	131	0.56	5382 (768-9475)	5270 (1581-9593)
BZ-S	b	155	0.55	5640 (1586-9604)	5365 (1055-9511)
XA-L	b	130	0.55	5764 (1059-9661)	4628 (815-9350)
XA-S	b	133	0.52	5364 (1069-9699)	4868 (645-9525)
FT-L	b	144	0.51	5680 (1243-9571)	5261 (889-9428)
FT-S	b	123	0.52	5870 (1542-9549)	4983 (813-9473)
CP-L	b	114	0.52	5705 (1190-9588)	5490 (940-9579)
CP-S	b	128	0.49	5712 (1312-9613)	4961 (841-9572)

Table S3 Gene introgression index of introduced *Amaranthus palmeri* in China from source populations in North and South American lineages based on microsatellite data.

Population	Introgression index	a (for North America)	b (for South America)
FZ-L	0.30	0.78	0.04
FZ-S	0.43	0.92	-0.02
CC-L	0.35	0.73	0.11
CC-S	0.27	0.80	-0.18
AY-L	0.07	0.77	0.17
AY-S	0.10	0.77	0.05
ZZ-L	0.41	0.84	0.05
ZZ-S	0.14	0.87	0.13
BZ-L	0.13	1.00	-0.12
BZ-S	0.27	0.72	-0.08
XA-L	0.00	0.96	0.12
XA-S	0.00	0.80	0.04
FT-L	0.11	0.84	0.13
FT-S	0.00	0.91	0.09
CP-L	0.08	0.85	-0.10
CP-S	0.00	0.91	-0.10

Table S4 Classification of *Amaranthus palmeri* populations (casual, naturalized, invasive, dispersal) across nine geographical regions based on establishment status and spread performance.

Geographical region code	Establishment status	Spread performance	Classification
DG	Transient	Confined to initial introduction sites (e.g., grain importation factories, ports)	Casual
FZ	Self-sustaining	Within 500 m of initial introduction sites	Naturalized
CC	Self-sustaining	Within 500 m of initial introduction sites	Naturalized
AY	Self-sustaining	Spread beyond 1000 m from initial introduction sites	Invasive
ZZ	Self-sustaining	Spread beyond 1000 m from initial introduction sites	Invasive
BZ	Self-sustaining	Spread beyond 1000 m from initial introduction sites	Invasive
XA	Self-sustaining	Spread >1000 m (thousands of meters) from initial introduction sites	Dispersal
FT	Self-sustaining	Spread >1000 m (thousands of meters) from initial introduction sites	Dispersal
CP	Self-sustaining	Spread >1000 m (thousands of meters) from initial introduction sites	Dispersal

As can be seen from the above table, *A. palmeri* populations in Dongguan (DG) were found only at initial introduction sites (e.g., grain importation factories and ports). These populations were transient (non-persistent) and failed to establish. We classified them as casual. In Fuzhou (FZ) and Changchun (CC), *A. palmeri* established self-sustaining populations that expanded locally from initial introduction sites. However, these populations remain confined to limited areas (<500 m from grain importation factories) and have not undergone significant spread. We classified them as naturalized. *A. palmeri* populations in Anyang (AY), Zhengzhou (ZZ), and Binzhou (BZ) established self-sustaining populations and spread rapidly beyond 1000 m from initial introduction sites within a few years. We classified them as invasive. *A. palmeri* populations in Xi'an (XA), Fengtai (FT), and Changping (CP) established self-sustaining populations and spread extensively, reaching distances of thousands of meters from initial introduction sites. We classified them as dispersal.

Table S5 cpDNA primers used in this study.

Intergenic regions	Primer name	Primer sequences (5'-3')	Tm/°C
petB-petD	petB-f<br>petD-r	CAATCCACTTTGACTCGTTTT<br>GGTTCACCAATCATTGATGGTTC	55
ropB-trnC	rpoB-f<br>trnC-r	ACAAAATCCTTCAAATTGTATCTGA<br>TTTGTTAATCAGGCGACACCCGG	55
petA-petJ	petA-f<br>psbJ-r	GGATTTGGTCAGGGAGATGC<br>ATGGCCGATACTACTGGAAGG	60

Notes: PCR amplification was carried out using a T100^{TM} Thermal Cycler (Bio-Rad, US) in a 20-μL reaction volume, consisting of 1 μL DNA (10–50 μM), 2 μL 10×TransStart Taq Buffer (Transgen, Beijing), 1.6 μL 2.5 mM dNTPs (Transgen, Beijing), 0.5 μL forward primer (10 μM), 0.5 μL reverse primer (10 μM), 0.2 μL TransStart Taq DNA Polymerase (Transgen, Beijing), and nuclease-free water. The PCR cycling conditions were an initial denaturation at 94ºC for 3 min and then 34 cycles of 94ºC for 30 s, 55ºC/60ºC (based on the Tm of primers) for 30 s, 72ºC for 1–2 min (based on the length of sequence), and ending with a final elongation step at 72ºC for 5 min. Samples were subsequently stored at 4ºC.

Table S6 *Amaranthus palmeri* microsatellite primers used in this study.

Primer name	Primer sequences (5'-3')	Tm/ºC	Size (bp)	Repeats	Labeled	Source
133462-F<br>133462-R	TGATGTTTCCC ATACACTAC<br>CCCTGGCATTT ATCTACT	52	180-210	$(TCA)_9$	5-FAM	Lab-designed
462991-F<br>462991-R	CTTGGGTTGAT TGTAGTGT<br>ATGGCAGGGA GGTTT	52	250-300	$(TC)_5$	5-FAM	Lab-designed
86259-F<br>86259-R	ATTGGGGATTT AGTTTAGG<br>GAGTAGTTTGA ACCAGGAA	52	250-300	$(TC)_7$	5-FAM	Lab-designed
7089-F<br>7089-R	ATCAAACCCTC TACTCT<br>ATCACAGCCGT CTATT	50	250-300	$(CTA)_6$	5-FAM	Lab-designed
125647-F<br>125647-R	ACTTGTTTATC CCTTTC<br>ATGTTGCGACT ATTGC	50	150-200	$(CT)_5$	5-FAM	Lab-designed
33422-F<br>33422-R	TTACCCAACGA GTTAGCC<br>CTCAATCAGTT CCGACAG	52	150-200	$(CTA)_6$	5-FAM	Lab-designed
GB-AMM-013-F<br>GB-AMM-013-R	TGAATCCTTAT GCGCCAC<br>GGAGCCCTGTC CTCATGT	52	130-180	$(AGG)_4$	5-ROX	Lee *et al.*, 2008
GB-AMM-032*-F<br>GB-AMM-032*-R	CTCCTCGGGAG AAGGTTG<br>TGTGTCCCAAT CCATCGT	55	130-170	$(GTG)_6$	5-ROX	Lee *et al.*, 2008
GB-AMM-051*-F<br>GB-AMM-051*-R	GAGGAGACTT GGTGGCCT<br>TCGGGAGCAAT GTAGCAC	55	200-250	$(AGA)_5$	5-ROX	Lee *et al.*, 2008
GB-AMM-099*-F<br>GB-AMM-099*-R	AAATTGACAAT GCGCAGC<br>TTCCTCACCAA AATTGCC	50	100-150	$(TCA)_{12}$,$(TCA)_5$	5-ROX	Lee *et al.*, 2008
GB-AMM-132*-F<br>GB-AMM-132*-R	AACTTTTGCCT CCTGCAA<br>TCAAATGCTGA TCCCAGG	55	100-150	$(AAG)_{17}$	5-ROX	Lee *et al.*, 2008
GB-AMM-136*-F<br>GB-AMM-136*-R	TCAGCAAAAC ATGATCAACAA<br>GTTGCTGCATT GGTGGTT	55	180-210	$(GAA)_6$, $(CCA)_6$	5-ROX	Lee *et al.*, 2008
